# Immunomagnetic Separation Combined with Polymerase Chain Reaction for the Detection of *Alicyclobacillus acidoterrestris* in Apple Juice

**DOI:** 10.1371/journal.pone.0082376

**Published:** 2013-12-03

**Authors:** Zhouli Wang, Jun Wang, Tianli Yue, Yahong Yuan, Rui Cai, Chen Niu

**Affiliations:** 1 College of Food Science and Engineering, Northwest A&F University, Yangling, Shaanxi, China; 2 College of Food Science and Engineering, XuChang University, XuChang, Henan, China; University of Houston, United States of America

## Abstract

A combination of immunomagnetic separation (IMS) and polymerase chain reaction (PCR) was used to detect *Alicyclobacillus acidoterrestris* (*A. acidoterrestris*) in apple juice. The optimum technological parameters of the IMS system were investigated. The results indicated that the immunocapture reactions could be finished in 60 min and the quantity of IMPs used for IMS was 2.5 mg/mL. Then the combined IMS-PCR procedure was assessed by detecting *A. acidoterrestris* in apple juice samples. The agarose gel electrophoresis results of 20 different strains showed that the IMS-PCR procedure presented high specificity to the *A*. acidoterrestris. The sensitivity of the IMS-PCR was 2×10^1^ CFU/mL and the total detection time was 3 to 4 h. Of the 78 naturally contaminated apple juice samples examined, the sensitivity, specificity and accuracy of IMS-PCR compared with the standardized pour plate method were 90.9%, 97.0% and 96.2%, respectively. The results exhibited that the developed IMS-PCR method will be a valuable tool for detecting *A. acidoterrestris* and improving food quality in juice samples.

## Introduction

*Alicyclobacillus acidoterrestris* (*A. acidoterrestris*), which contains ω-fatty acids as the major membrane fatty acid component, is a thermophilic, aciduric, gram-positive, rod-shaped, spore-forming and nonpathogenic bacterium [1–3]. As it can survive in highly acidic environments of pH 2.5-6.0 at temperatures of 25-60 °C, and its spore can germinate and proliferate in acidic fruit juices or beverages, *A. acidoterrestris* is extremely difficult to be inactivated completely by conventional pasteurization [4,5]. *A. acidoterrestris*-related spoilage is characterized by the formation of medicinal or antiseptic off-odors. However, the spoiled apple juice usually looks normal, without obvious pH change, gas production and evident sediment formation, which make it hard to detect by visible inspection [6,7]. 

Traditional methods for detecting *A. acidoterrestris* are based on selective enrichment and subsequent culture on a selective medium, which are laborious and take up to 3-5 days to give a positive result [8]. To expeditiously detect this bacterium in food samples, a number of rapid methods have been developed, including polymerase chain reaction (PCR), Enzyme-linked immunosorbent assay (ELISA), Fourier transform infrared (FT-IR), electronic nose and 16S rDNA gene sequence analysis [4,9–11]. However, the complex background interference of the food components and the non-target flora has a negative impact on the detection of target bacteria [7,12]. In addition, the target bacteria are generally present in very low numbers, or even just a few cells. These detection techniques are usually used following cultivation processes, which increases the detection time [11,12]. Therefore, a reliable isolation and enrichment pre-treatment procedure is crucial for rapid and sensitive detection of *A. acidoterrestris* in fruit juices.

Immunomagnetic separation (IMS) technique, which is time-saving and shows high specificity for the separation and concentration of target organisms, has been evaluated as a successful pre-treatment for the detection [13]. The immunomagnetic nanoparticles (IMPs) coated with specific antibody were used to capture the target organism. The whole bacteria-bead complexes were separated from the sample using a magnetic field for further analysis [14–16]. At present, IMS has been successfully applied in conjunction with PCR, ELISA and others to further increase their detection sensitivity for foodborne pathogens [14,17,18]. 

In this study, the technological parameters of IMS system were optimized for separation and enrichment of *A. acidoterrestris* from apple juice samples. Then IMS technique combining with PCR (IMS-PCR) was used to detect *A. acidoterrestris*. The detection limit and specificity of the IMS-PCR method were determined. The performance of the developed IMS-PCR was further evaluated by detecting of *A. acidoterrestris* in naturally contaminated apple juice samples.

## Materials and Methods

### Bacterial strains

The strains used in this study were listed in Table 1. All the strains were incubated under their best conditions. Five hundred milliliters of each bacteria culture was centrifuged at 3622 g for 15 min at 4 °C to collect cells from the culture solution. The pellets were washed twice by resuspending in sterilized water and centrifuging as before. The final pellets were suspended in sterilized water and the cell concentration of the suspension was approximately 10^4^ CFU/mL, which was determined by the direct plating method. The suspension was then stored at 4 °C.

**Table 1:** List of strains and the specificity testing of them using IMS-PCR.

Strain	Species	Medium	Tem*	Strain	Species	Medium	Tem
446	*A. acidocaldariuss **^a^***	402 **^*A*^**	60 °C	14955	*A. pomorum **^a^***	402	45 °C
448	*A. acidocaldarius **^a^***	402	60 °C	17614	*A. sendaiensis **^a^***	402	50 °C
2498	*A. acidoterrestris **^a^***	402	45 °C	17975	*A. contaminans **^a^***	13	55 °C
3922	*A. acidoterrestris **^a^***	402	45 °C	17978	*A. fastidiosus **^a^***	13	45 °C
3923	*A. acidoterrestris **^a^***	402	45 °C	AAT 13	*A. acidoterrestris **^b^***	402	45 °C
3924	*A. acidoterrestris **^a^***	402	45 °C	YL-5	*A. contaminans **^c^***	402	45 °C
4006	*A. cycloheptanicus **^a^***	402	45 °C	YL-3	*B. subtilis **^c^***	402	45 °C
12489	*A. hesperidum **^a^***	402	50 °C	LC-8	*B. ginsengihumi **^c^***	402	45 °C
13609	*A. herbarius **^a^***	13 **^*B*^**	55 °C	BS-2	*B. ginsengihumi **^c^***	402	45 °C
14558	*A. acidiphilus **^a^***	402	45 °C	C-18	*A. acidoterrestris **^c^***	402	45 °C

* Tem: Temperature.

Various superscripts in lowercase indicate the sources of strains. ***^a^*** DSMZ: German Resource Centre for Biological Material; ***^b^*** AAT: Food Research Laboratories, Mitsui Norin Co., Ltd., Tokyo, Japan; ***^c^*** NWSUAF: College of Food Science and Engineering, Northwest A&F University.

Various superscripts in uppercase indicate the enrichment medium of strains. ***^A^*** 402 medium: 0.2 g ammonia sulfate, 0.25 g calcium chloride, 0.5 g magnesium sulfate, 2.0 g yeast extract, 5.0 g glucose, and 3.0 g monopotassium phosphate per liter of deionized water (pH 4.0). ***^B^*** 13 medium: 2.0 g yeast extract, 0.2 g ammonia sulfate, 0.5 g magnesium sulfate, 0.25 g calcium chloride, 0.6 g monopotassium phosphate, 1.0 g glucose, 0.01 g manganese sulfate per liter of deionized water (pH 3.0-4.0).

### Optimization of the immunomagnetic separation system

IMPs were prepared by immobilizing the specific polyclonal anti-*Alicyclobacillus* IgG antibody onto the surface of the amino-silane modified magnetic nanoparticles (SMNPs) as recommended in our previous study [4,16]. The obtained IMPs were resuspended in 0.01 mol/L phosphate buffer sodium (PBS, pH 7.4) plus 5% bovine serum albumin (BSA) and 0.05 % Tween-20 and stored at 4 °C for use. The immunocapture time and the quantity of IMPs used for the separation of *A. acidoterrestris* in apple juice samples were optimized for maximal immunocapture efficiency.

The fresh culture of *A. acidoterrestris* ten-fold diluted by reconstituted apple juice (15 °Brix) was used as the spiked samples. Two milliliters of the spiked sample (2×10^4^ CFU/mL) were transferred to microcentrifuge tubes, and 5 mg of IMPs were added into the suspensions. The mixtures were incubated at 37 °C for different times (10, 30, 60 and 120 min). Besides, the samples containing different concentrations of *A. acidoterrestris* (from 2×10^1^ CFU/mL to 2×10^6^ CFU/mL) were separated by different quantities of IMPs (2.5, 5, 10 and 20 mg) and the mixtures were incubated at 37 °C for 60 min. Then the bacteria-beads complexes were collected by magnetic force, washed three times and resuspended in 200 μL PBS. The obtained samples (100 μL) were streaked on yeast extract starch glucose (YSG) agar and incubated at 45°C for 3 days prior to enumeration. The immunocapture efficiency (CE) was defined as the percentage of the total bacteria present in suspension that were captured by the IMPs.

### DNA extraction and PCR detection

**Figure 1:**
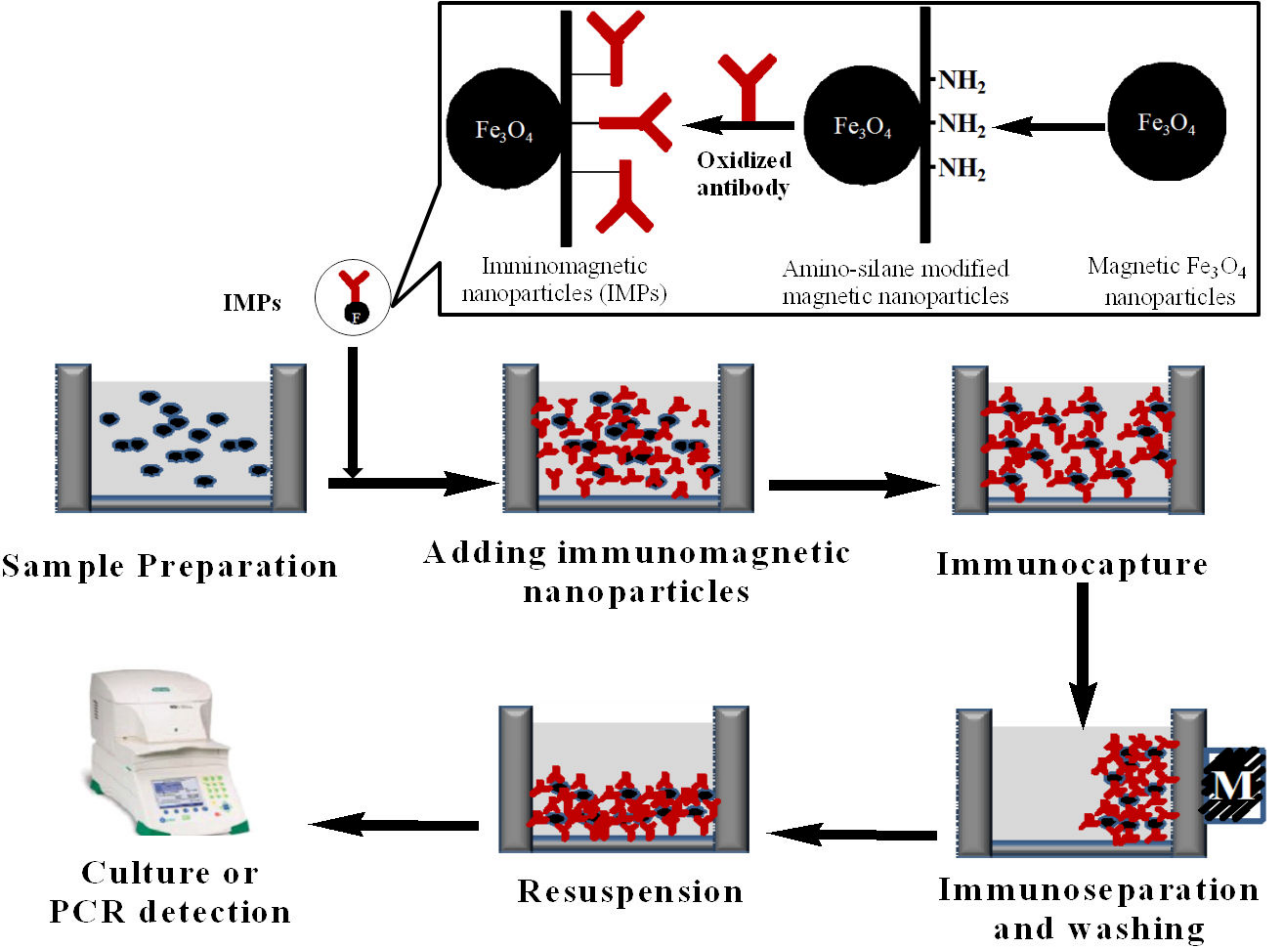
The flow chart for the isolation and detection of *A. acidoterrestris* by IMS-PCR.

The flow chart for the isolation and detection of *A. acidoterrestris* by IMS-PCR was shown in Figure 1. After the separation and enrichment of target bacteria, aliquots (50 μL) of the complexes were collected using a magnet and resuspended in 50 μL of Tris-HCl-EDTA buffer (TE, pH 8.3). The mixture was boiled for 10 min and centrifuged at 4,000×g for 5 min (4 °C). The supernatant was collected and stored at -20 °C for PCR amplification.

The PCR detection of *A. acidoterrestris* was performed with the primers that had been developed and validated [19]. The sequences of the primers were Forward 5’-ACGGGTAGGCATCTACTTGT-3’ for Ba 190F and Reverse 5’-AGGAGCTTTCCACTCTCCTTGT-3’ for Ba 490R. The PCR reaction mixture contained 25 μL Premix Taq (20 mM Tris-HCl, pH 8.3, 100 mM KCl, 3 mM MgCl_2_, 0.4 mM dNTP Mixture, 1.25 units /25 μL TaKaRa Taq DNA polymerase, Takara Biotechnology Co., Ltd), 1 μL each primer (10 μM), 13 μL distilled water and 10 μL template. PCR assays were conducted in a DNA Engine Peltier Cycler (Bio-RAD, Hercules, Calif., U.S.A.) with the following thermal profile: initial denaturation at 94 °C for 4 min, followed by 35 cycles of denaturation at 94 °C for 30 s, annealing at 50 °C for 30 s, extension at 72 °C for 30 s, and final extension at 72 °C for 5 min. Amplified PCR products were separated in a 1% (w/v) agarose gel electrophoresis (AGE) and stained with ethidium bromide.

### Sensitivity of the IMS-PCR assay

In order to determine the detection limit of the IMS-PCR assay, 1 mL ten-fold dilutions of *A. acidoterrestris* (from 2×10^0^ CFU/mL to 2×10^5^ CFU/mL) was added into 9 mL sterilized water and reconstituted apple juice (15 °Brix), respectively. The pure culture samples and artificially contaminated apple juice were detected by the IMS-PCR. The PCR analysis without IMS enrichment was considered as a control. The concentration of the highest dilution giving a specific band was defined as the detection limit.

### Specificity of the developed IMS-PCR

As shown in Table 1, twenty strains including fourteen standard strains of *Alicyclobacillus* spp. and six isolates were used to determine the specificity of IMS-PCR. The pellets of bacteria were suspended in sterilized water. The final cell concentration was approximately 10^4^ CFU/mL. The samples were then subjected to the IMS-PCR procedure.

### Detection of *A. acidoterrestris* in apple juice samples

A total of 78 naturally contaminated apple juice samples, which were obtained from Shaanxi Haisheng Fresh Fruit Juice Co., Ltd, were used to evaluate the effectiveness of the IMS-PCR. The apple juice samples were diluted with sterilized water and the soluble solid content was about 15 °Brix. Then 2 mL of each sample was enriched and detected by the IMS-PCR procedure. All of the apple juice samples were also detected according to the protocol of a standardized pour plate method issued by the Japan Fruit Juice Association [20,21].

## Results

### Optimization of the immunomagnetic separation system

**Figure 2:**
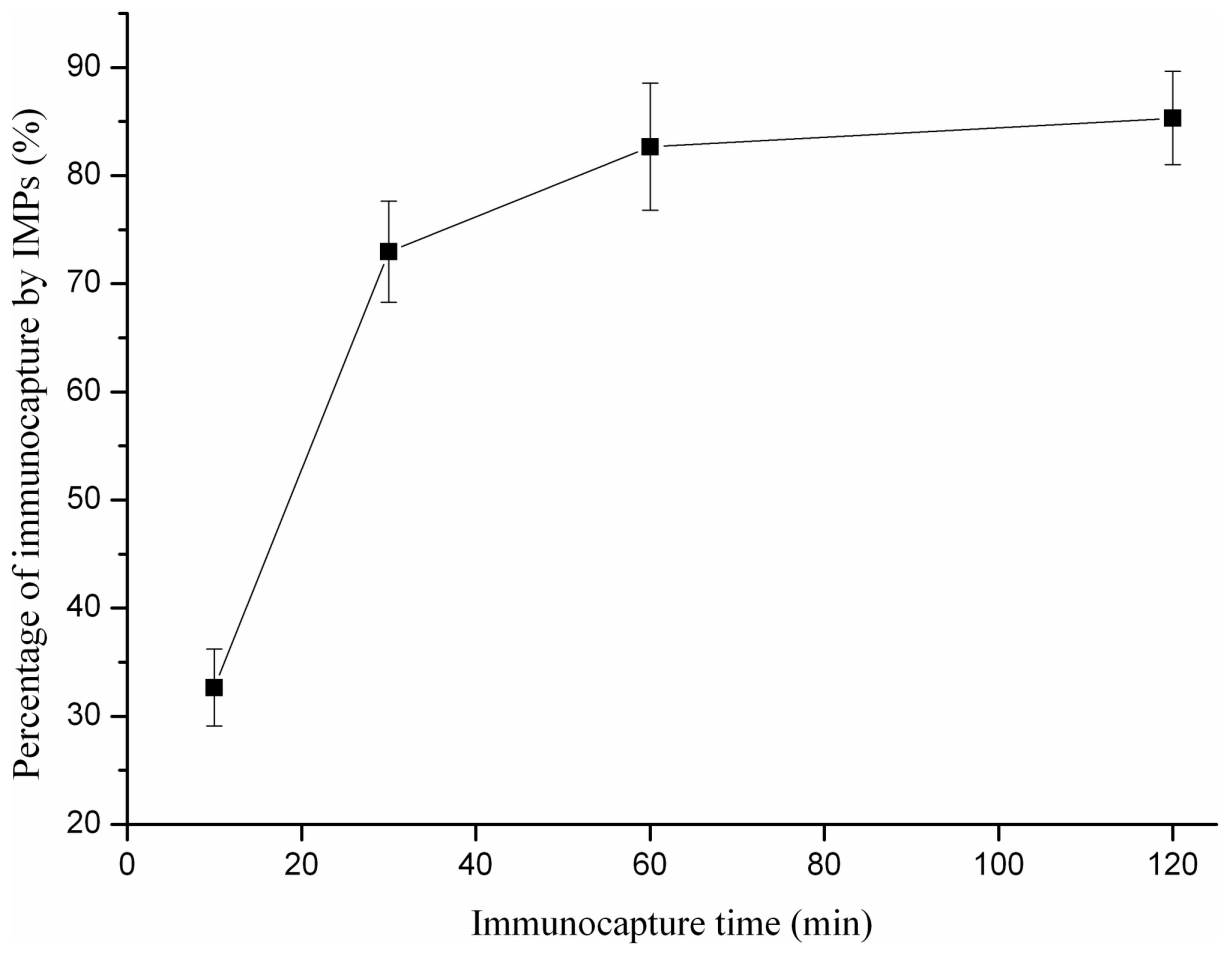
The effect of immunocapture time on the separation of *A. acidoterrestris* in apple juice.

For a rapid and efficient isolation and enrichment, the effects of coupling time and the quantity of IMPs on the immunocapture of bacteria were investigated. As shown in Figure 2, the coupling time had a significant impact on the immunocapture of *A. acidoterrestris* in apple juice. With the coupling time increased from 10 to 30 min, CE of *A. acidoterrestris* rose dramatically from 32.6% to 72.9%. Then the CE grew slightly to 82.6% in the following 30 min. As the coupling time continuously increased to 120 min, there was no significant change in the percentage of recovery. The separation of *A. acidoterrestris* by IMPs reached the adsorption equilibrium in 60 min and the longer reaction time did not increase the capture efficiency significantly. Therefore, the optimal immunocapture time for *A. acidoterrestris* was selected as 60 min.

**Figure 3:**
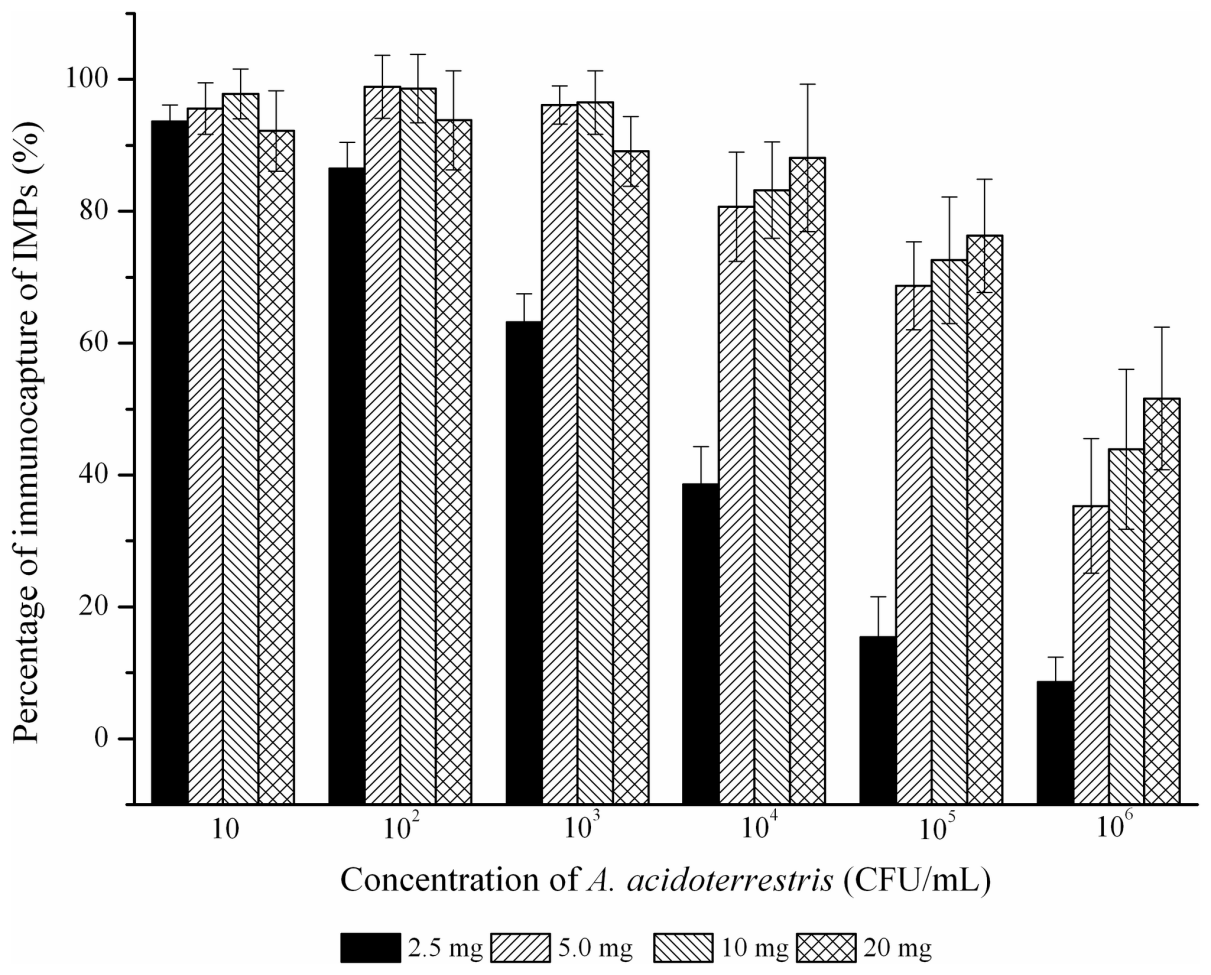
The effect of quantity of IMPs on the immunocapture of *A. acidoterrestris*.

The effects of the quantity of IMPs on the immunocapture of *A. acidoterrestris* were shown in Figure 3. When 2.5 mg of IMPs were used for separation, the immunocapture efficiency for *A. acidoterrestris* (less than 10^3^ CFU/mL) was greater than 80%, and then the recovery proportion of the bacteria population was significantly decreased with the increase of *A. acidoterrestris* concentration. As the IMPs dosage increased to 5 mg, the recovery percentage for *A. acidoterrestris* improved obviously under the bacteria concentration ranging from 10^2^ to 10^6^ CFU/mL in comparison with the usage of 2.5 mg. More than 80% of *A. acidoterrestris* were captured by 5 mg IMPs when the bacteria concentration was less than or equal to 10^4^ CFU/mL. When the quantity of IMPs rose to 10 or 20 mg, the recovery percentage of *A. acidoterrestris* did not improve significantly at any bacteria concentration examined (from 10 to 10^6^ CFU/mL). For overall economics and efficiency, 5 mg of IMPs were used for the capture of *A. acidoterrestris* cells from 2 mL test samples in all future experiments (2.5 mg/mL).

### Sensitivity of the developed IMS-PCR assay

**Figure 4:**
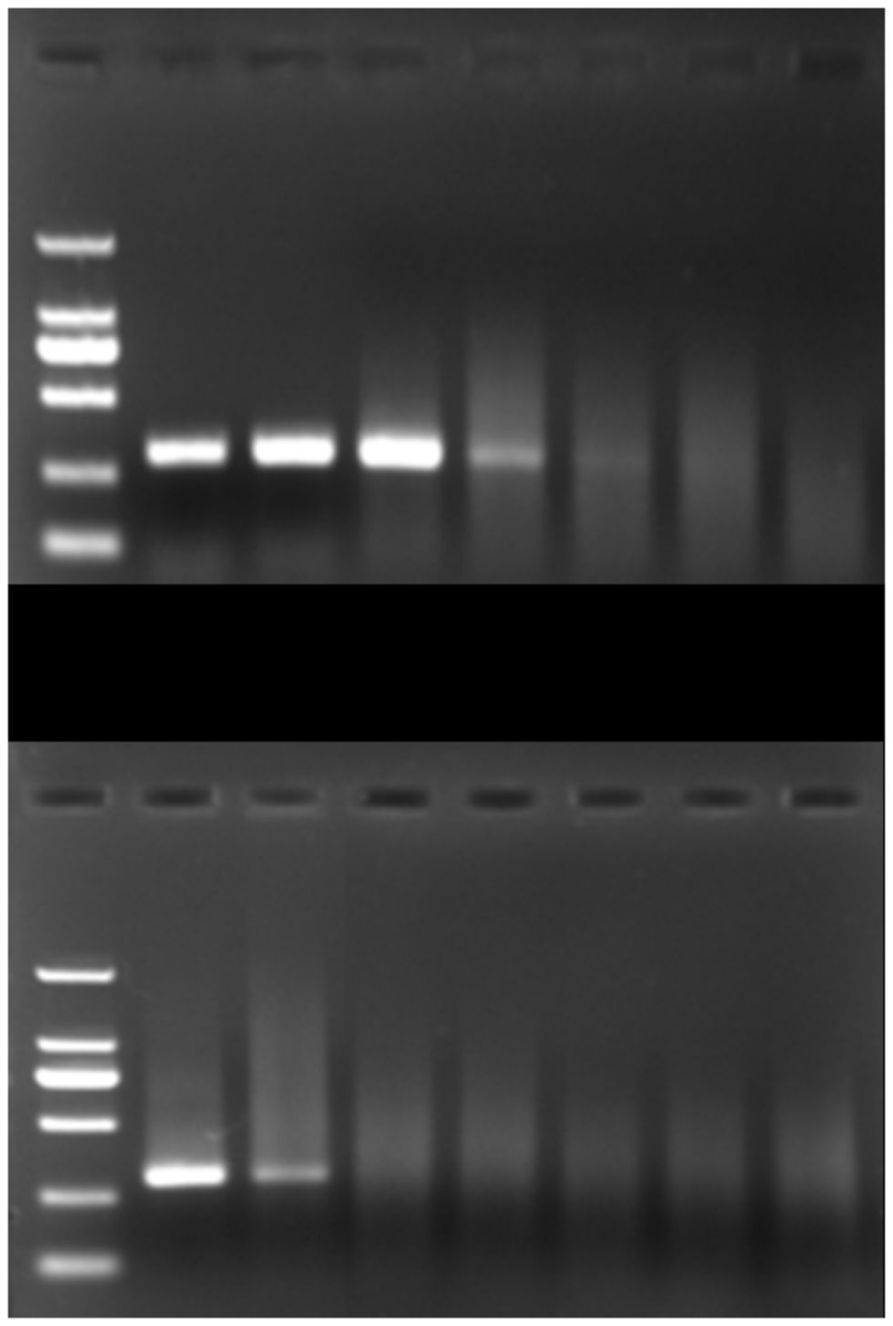
Comparison of the ability of IMS-PCR (a) and PCR (b) to detect *A. acidoterrestris* in apple juice. Lane M, DNA markers (100 bp); lines 1-6, 10^4^, 10^3^, 10^2^, 10^1^, 10°, 10^-1^ CFU/mL; line 7, negative control.

The artificially contaminated apple juice samples with different concentrations (2×10^-1^ CFU/mL to 2×10^4^ CFU/mL) were analyzed by the IMS-PCR and PCR. The results of AGE were shown in Figure 4. The band of the IMS-PCR was clear to be observed when the *A. acidoterrestris* concentration was 2×10^1^ CFU/mL in apple juice sample (Figure 4a). However, the band of the PCR without IMS was not present, even at the concentration up to 2×10^2^ CFU/mL (Figure 4b). The results demonstrated that the detection limit of the IMS-PCR (2×10^1^ CFU/mL) was two orders of magnitude lower than that of the direct PCR (2×10^3^ CFU/mL), i.e., low populations of *A. acidoterrestris* in apple juice could be more accurately detected by IMS-PCR than direct PCR. The lower limit of IMS-PCR obtained from apple juice sample was consistent with the results from pure culture (data not shown), which indicated that apple juice ingredients had little influence on bacteria separation and determination.

### Specificity of the IMS-PCR assay

**Figure 5:**
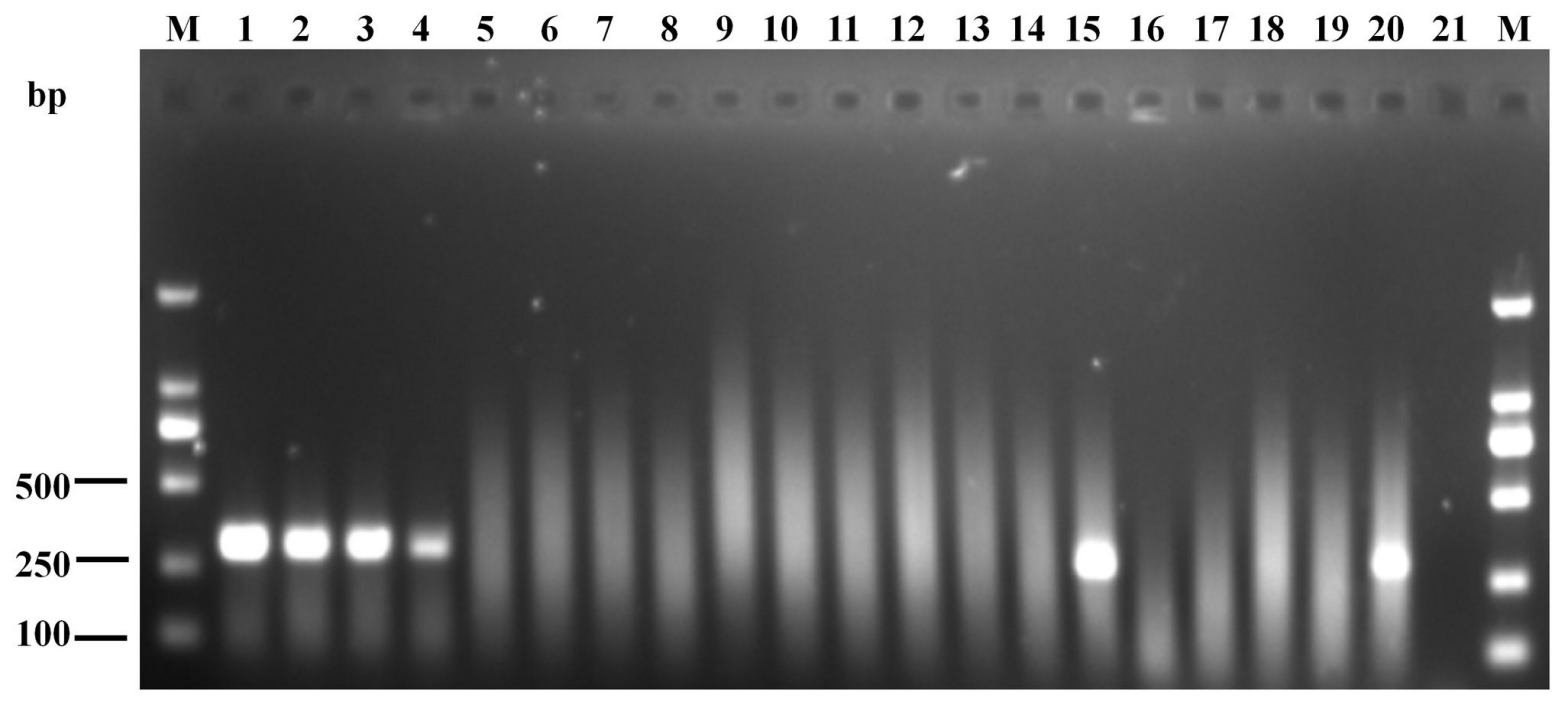
Specificity of the IMS-PCR assay. Lane M, DNA markers (100 bp); lines 1-4, *A. acidoterrestris* (DSM 2498, DSM 3922, DSM 3923, DSM 3924); lines 5-6, *A. acidocaldarius* (DSM 446, DSM 448); line 7, *A. cycloheptanicus* (DSM 4006); line 8, *A. hesperidum* (DSM 12489); line 9, *A. herbarius* (DSM 13609); line 10, *A. acidiphilus* (DSM 14558); line 11, *A. pomorum* (DSM 14955); line 12, *A. sendaiensis* (DSM 17614); line 13, *A. contaminans* (DSM 17975); line 14, *A. fastidiosus* (DSM 17978); line 15, *A. acidoterrestris* (AAT 13); line 16, *A. contaminans* (YL-5); line 17, *B. subtilis* (YL-3); lines 18-19, *B. ginsengihumi* (LC-8, BS-2); line 20, *A. acidoterrestris* (C-18); line 21, negative control.

To check the specificity of the IMS-PCR assay, samples from all strains listed in Table 1 were enriched by IMS and subjected to 35 cycles of amplification. Assays were performed in triplicate, and a representative PCR chart was shown in Figure 5. According to the output chart, *A. acidoterrestris* (DSM 2498, DSM 3922, DSM 3923, DSM 3924 and AAT 13) produced only an intense band (298 bp) with primer annealing at 50 °C. A similar band also appeared in the lane spotted with *A. acidoterrestris* (C-13), which was isolated from concentrated apple juice. On the other hand, no PCR product was detected from other tested bacteria. The results suggested that under proper stringent conditions, the combination of immunocapture procedure and the sequences of the oligonucleotide primers used in the study were distinctive enough to distinguish *A. acidoterrestris* from others.

### Detection of *A. acidoterrestris* in apple juice

Seventy-eight naturally contaminated apple juice samples were assayed by both IMS-PCR procedure and the standardized pour plate method. As shown in Table 2, ten samples (12.82% of 78 samples) were identified positive, while 65 samples (83.33%) were negative by both IMS-PCR and isolation with YSG agar. One apple juice sample identified as *A. acidoterrestris* positive by YSG agar was negative using IMS-PCR. Two samples dispalyed positive by IMS-PCR, but were *A. acidoterrestris* negative by culture. The results from the standardized pour plate method and IMS-PCR were consistent for the detection of *A. acidoterrestris* in 75 apple juice samples. As compared to the standardized pour plate method performed concurrently with the same samples, the sensitivity, specificity and accuracy of the developed IMS-PCR procedure were 90.9%, 97.0% and 96.2%, respectively. The results indicated that these two detection methods showed no statistically significant differences and the proposed IMS-PCR can be effectively used to detect *A. acidoterrestris* in apple juice.

**Table 2:** Comparison of IMS-PCR and the standardized pour plate method (YSG agar) in apple juice samples detection.

YSG agar	IMS-PCR		Total	Sensitivity **^*a*^** (100 %)	Specificity **^*b*^** (100 %)	Accuracy **^*c*^** (100 %)
	Positive	Negative				
Positive	10	1	11	90.9%	97.0%	96.2%
Negative	2	65	67			
Total	12	66	78			

*a* Sensitivity = TP/(TP+FN) ×100% (TP: True positive, FN: False negative).

*b* Specificity = TN/(TN+FP)×100% (TN: True negative, FP: False positive).

*c* Accuracy = (TP+TN)/(TP+TN+FP+FN)×100%.

## Discussions

For the IMS procedure, the entire surfaces of captured bacterial cells were covered by IMPs due to their smaller sizes compared to bacterial cells [22]. An excessive quantity of IMPs present on the cell surface is desirable for cell separation. However, this may negative effects on the DNA extraction efficiency and the detection limit of PCR [23]. Herein, 5 mg of IMPs was used for the isolation and enrichment of *A. acidoterrestris* in 2 mL samples system (2.5 mg/mL). Although the recovery proportion of IMS was actually lost with the concentration of bacteria more than 10^4^ CFU/mL, the total numbers of isolated bacteria cells still increased and could meet the requirement of detection. Furthermore, high numbers of *A. acidoterrestris* would never be encountered in commercialized apple juice, and when the concentration was above 10^5^ CFU/mL, the juice samples can be detected directly by PCR, ELISA or other methods [4,9,19].

With the advantage of much faster than using vectors and only needing very small amount of target DNA, PCR technique is used as a simple and rapid detection method for high sample throughput [24,25]. In this study, PCR procedure was combined with IMS for the detection of *A*. acidoterrestris in apple juice. The detection limit of IMS-PCR was 2×10^1^ CFU/mL and the testing process took about 3-4 h. Compared with the reported molecular methods for *Alicyclobacillus* spp., IMS-PCR procedure has improved the sensitivity and shortened the total analysis time. K. Yamazaki et al [19] have reported a reverse transcription PCR capable of detecting 10^4^ CFU/mL of *A*. acidoterrestris. Conner et al [9] and Luo et al [11] have developed a real-time PCR for detection of *Alicyclobacillus* spp. in juice products, with a detection limit less than 100 cells.

To further investigate the performance of the testing system, the naturally contaminated apple juice samples were detected by IMS-PCR. Compared with the standardized pour plate method, 2 of 67 PCR assays were judged to be false negative, and 1 of 11 showed false positive. There are several factors contributing to the difference of the detection results. For the plate method, other *Alicyclobacillus* spp. not just *A. acidoterrestris* could be cultivable on the YSG agar [26]. PCR detects the total DNA of microorganisms no matter if they are alive or not. *A. acidoterrestris* cells that are inactive in samples could be detected as positive, which is also a likely explanation for the observed discrepancy between culture and IMS-PCR methods [23]. This paper was a tentative exploration on the isolation and detection of *A. acidoterrestris* by IMS-PCR. A follow-up study will be needed to combine the IMS procedure with the real-time PCR assay or some modified PCR approach (such as batch-stamps and barcodes) to minimize the risk of PCR contamination [27,28]. 

In conclusion, a novel IMS-PCR procedure was developed for the detection of *A. acidoterrestris* in apple juice. The method showed high specificity for *A. acidoterrestris*. The testing process was finished in 3-4 h and the detection limit of IMS-PCR was 2×10^1^ CFU/mL. As a rapid, specific and sensitive detection method, the IMS-PCR procedure proposed in this study has great potential for detecting *A. acidoterrestris* in the fruit juice or beverage industry.

## References

[1] Siegmund B, Pöllinger-Zierler B (2007) Growth behavior of off-flavor-forming microorganisms in apple juice. J Agric Food Chem 55: 6692-6699. doi:10.1021/jf070524j. PubMed: 17616209. 17616209

[2] Danyluk MD, Friedrich LM, Jouquand C, Goodrich-Schneider R, Parish ME et al. (2011) Prevalence, concentration, spoilage, and mitigation of Alicyclobacillus spp. in tropical and subtropical fruit juice concentrates. Food Microbiol 28: 472-477. doi:10.1016/j.fm.2010.10.008. PubMed: 21356453. 21356453

[3] Zhang J, Yue T, Yuan Y (2013) *Alicyclobacillus* Contamination in the Production Line of Kiwi Products in China. PLOS ONE 8: e67704. doi:10.1371/journal.pone.0067704. PubMed: 23844069. 23844069 PMC3699629

[4] Wang Z, Yue T, Yuan Y, Cai R, Guo C et al. (2012) Development of polyclonal antibody based indirect Enzyme Linked Immunosorbent Assay for the detection of *Alicyclobacillus* strains in apple juice. J Food Sci, 77: M643-M649. PubMed: 23106215. 23106215 10.1111/j.1750-3841.2012.02961.x

[5] Wang Z, Yue T, Yuan Y, Cai R, Niu C et al. (2013) Development and evaluation of an immunomagnetic separation-ELISA for the detection of *Alicyclobacillus* spp. in apple juice. Int J Food Microbiol 166: 28-33. doi:10.1016/j.ijfoodmicro.2013.06.015. PubMed: 23827805. 23827805

[6] Perez-Cacho PR, Rouseff R (2008) Processing and storage effects on orange juice aroma: A review. J Agric Food Chem 56: 9785-9796. doi:10.1021/jf801244j. PubMed: 18828595. 18828595

[7] Smit Y, Cameron M, Venter P, Witthuhn RC (2011) *Alicyclobacillus* spoilage and isolation–A review. Food Microbiol 28: 331-349. doi:10.1016/j.fm.2010.11.008. PubMed: 21356436. 21356436

[8] Henczka M, Djas M, Filipek K (2013) Optimisation of a direct plating method for the detection and enumeration of *Alicyclobacillus* *acidoterrestris* spores. J Microbiol Methods 92: 1-8. doi:10.1016/j.mimet.2012.10.007. PubMed: 23098919. 23098919

[9] Connor CJ, Luo H, McSpadden Gardener BB, Wang HH (2005) Development of a real-time PCR-based system targeting the 16S rRNA gene sequence for rapid detection of *Alicyclobacillus* spp. in juice products. Int J Food Microbiol 99: 229-235. doi:10.1016/j.ijfoodmicro.2004.08.016. PubMed: 15808357. 15808357

[10] Concina I, Bornšek M, Baccelliere S, Falasconi M, Gobbi E et al. (2010) *Alicyclobacillus* spp.: Detection in soft drinks by Electronic Nose. Food Research International 43: 2108-2114. doi:10.1016/j.foodres.2010.07.012.

[11] Luo H, Yousef AE, Wang HH (2004) A real-time polymerase chain reaction-based method for rapid and specific detection of spoilage Alicyclobacillus spp. in apple juice. Lett Appl Microbiol 39: 376-382. doi:10.1111/j.1472-765X.2004.01596.x. PubMed: 15355542. 15355542

[12] Patel P (2000) A review of analytical separation, concentration and segregation techniques in microbiology. Journal of Rapid Methods and Automation in Microbiology 8: 227-248. doi:10.1111/j.1745-4581.2000.tb00326.x.

[13] Španová A, Rittich B, Horák D, Lenfeld J, Prodělalová J et al. (2003) Immunomagnetic separation and detection of *Salmonella* cells using newly designed carriers. Journal of Chromatography A 1009: 215-221. doi:10.1016/S0021-9673(03)00431-X. PubMed: 13677662. 13677662

[14] Malkova K, Rauch P, Wyatt G, Morgan M (1998) Combined immunomagnetic separation and detection of *Salmonella* *enteritidis* in food samples. Food and Agricultural Immunology 10: 271-280. doi:10.1080/09540109809354990.

[15] Jeníková G, Pazlarová J, Demnerová K (2000) Detection of *Salmonella* in food samples by the combination of immunomagnetic separation and PCR assay. International Microbiology 3: 225-229. PubMed: 11334305. 11334305

[16] Wang Z, Yue T, Yuan Y, Cai R, Niu C et al. (2013) Preparation of immunomagnetic nanoparticles for the separation and enrichment of *Alicyclobacillus* spp. in apple juice. Food Research International 54: 302-310. doi:10.1016/j.foodres.2013.07.021.

[17] Yang Z-Y, Shim W-B, Kim K-Y, Chung D-H (2010) Rapid detection of *Enterotoxigenic* *Clostridium* *perfringens* in meat samples using immunomagnetic separation Polymerase Chain Reaction (IMS- PCR). J Agric Food Chem 58: 7135-7140. doi:10.1021/jf1009654. PubMed: 20507064. 20507064

[18] Liandris E, Gazouli M, Andreadou M, Sechi LA, Rosu V et al. (2011) Detection of Pathogenic Mycobacteria Based on Functionalized Quantum Dots Coupled with Immunomagnetic Separation. PLOS ONE 6: e20026 PubMed: 21637746. 21637746 10.1371/journal.pone.0020026 PMC3103498

[19] Yamazaki K, Teduka H, Inoue N, Shinano H (1996) Specific primers for detection of *Alicyclobacillus* *acidoterrestris* by RT‐PCR. Lett Appl Microbiol 23: 350-354. doi:10.1111/j.1472-765X.1996.tb00206.x. PubMed: 8987718. 8987718

[20] Wang Y, Yue T, Yuan Y, Gao Z (2010) Isolation and identification of thermo-acidophilic bacteria from orchards in China. Journal of Food Protection ® 73: 390-394 10.4315/0362-028x-73.2.390 20132690

[21] Yokota Akira, Tateo Fujii Goto K (2007) Alicyclobacillus:Thermophilic Acidophilic Bacilli. Springer: 64-66.

[22] Zhao X, Hilliard LR, Mechery SJ, Wang Y, Bagwe RP et al. (2004) A rapid bioassay for single bacterial cell quantitation using bioconjugated nanoparticles. Proc Natl Acad Sci U S A 101: 15027-15032. doi:10.1073/pnas.0404806101. PubMed: 15477593. 15477593 PMC524056

[23] Yang H, Qu L, Wimbrow AN, Jiang X, Sun Y (2007) Rapid detection of *Listeria* *monocytogenes* by nanoparticle-based immunomagnetic separation and real-time PCR. Int J Food Microbiol 118: 132-138. doi:10.1016/j.ijfoodmicro.2007.06.019. PubMed: 17716768. 17716768

[24] Saiki RK, Scharf S, Faloona F, Mullis KB, Horn GT et al. (1985) Enzymatic amplification of b-globin genomic sequences and restriction site analysis for diagnosis of sickle cell anemia. Science 230: 1350-1354. doi:10.1126/science.2999980. PubMed: 2999980. 2999980

[25] Saiki RK, Gelfand DH, Stoffel S, Scharf SJ, Higuchi R et al. (1988) Primer-directed enzymatic amplification of DNA with a thermostable DNA polymerase. Science 239: 487-491. doi:10.1126/science.2448875. PubMed: 2448875. 2448875

[26] Murray MB, Gurtler JB, Ryu J-H, Harrison MA, Beuchat LR (2007) Evaluation of direct plating methods to enumerate *Alicyclobacillus* in beverages. Int J Food Microbiol 115: 59-69. doi:10.1016/j.ijfoodmicro.2006.10.025. PubMed: 17270301. 17270301

[27] McCloskey ML, Stöger R, Hansen RS, Laird CD (2007) Encoding PCR products with batch-stamps and barcodes. Biochem Genet 45: 761-767. doi:10.1007/s10528-007-9114-x. PubMed: 17955361. 17955361

[28] Fu Z, Rogelj S, Kieft TL (2005) Rapid detection of *Escherichia* *coli* O157: H7 by immunomagnetic separation and real-time PCR. Int J Food Microbiol 99: 47-57. doi:10.1016/j.ijfoodmicro.2004.07.013. PubMed: 15718028. 15718028

